# The Healthy Lifestyles Programme (HeLP) — An Overview of and Recommendations Arising from the Conceptualisation and Development of an Innovative Approach to Promoting Healthy Lifestyles for Children and Their Families 

**DOI:** 10.3390/ijerph120101003

**Published:** 2015-01-20

**Authors:** Jenny Lloyd, Katrina Wyatt

**Affiliations:** Institute for Health Research, University of Exeter Medical School, Veysey Building, Salmon Pool Lane, Exeter, Devon, EX2 4SG, UK; E-Mail: K.M.Wyatt@exeter.ac.uk

**Keywords:** engagement, behaviour change, supportive contexts, interactive drama, trusting relationships

## Abstract

Despite the rise in childhood obesity, there remains a paucity of evidence for effective interventions that engage children and parents sufficiently to make and sustain lifestyle behaviour change. The Healthy Lifestyles Programme (HeLP) is a school-located obesity prevention programme, which has been developed with teachers, families and healthcare professionals. The underpinning assumption in the development of HeLP was to take a relational approach to changing behaviour, building relationships with the schools, children and their families to create supportive environments for healthy lifestyle choices. Thus, HeLP was conceptualised as a complex intervention within a complex system and developed as a dynamic, evolving set of processes to support and motivate children towards healthy behaviours. The delivery methods used are highly interactive and encourage identification with and ownership of the healthy lifestyle messages so that the children are motivated to take them home to their parents and effect change within the family. We have good evidence that HeLP engages schools and children such that they want to participate in the Programme. Results from an exploratory trial showed that the Programme is feasible and acceptable and has the potential to change behaviours and affect weight status. This paper presents an overview of and recommendations arising from the conceptualization; development and evaluation of the Healthy Lifestyles Programme as part of a special issue focusing on novel approaches to the global problem of childhood obesity.

## 1. Introduction

Childhood obesity is one of the most serious public health challenges of the 21st century [1]. The problem is global and is steadily affecting many low- and middle-income countries, particularly in urban settings. Globally, in 2010 the number of overweight children under the age of five was estimated to be over 42 million. Close to 35 million of these are living in developing countries [1]. North America, Europe and parts of the Western Pacific have the highest prevalence of overweight children (approximately 20%–30%) with North America and Western Europe having the greatest annual increases in obesity [2]. Furthermore, the prevalence of overweight school children in several countries undergoing economic growth, such as Brazil, Chile, Mexico and Egypt has reached a level comparable to those in fully industrialised countries [2]. 

The Foresight Obesity report, which forecasted obesity prevalence among the general population in the UK from current trends suggested that by 2050, 60% of men and 50% of women will be obese, with growing disparities in risk for obesity by ethnicity and socio-economic status projected [3]. Data from England’s National Childhood Measurement Programme (NCMP) for 2012–2013 showed that 22.2% of children entering primary school were overweight or obese, rising to 33.3% when they leave primary school. Of these combined percentages, 9.3% and 18.9% were categorised as obese at Reception and Year 6, respectively [4]. Recent UK trends suggest that the rate of increase in childhood obesity may have slowed [5], however the prevalence rates are still high and significantly higher than before 1980 [6]. Inequalities among children continue to increase, with the highest prevalence of overweight and obesity among those from poorer backgrounds [5]. Until recently, evidence on the incidence of overweight and obesity by age group has been limited, resulting in a lack of explicit guidance in choice of target population for obesity prevention [7]. However, data from a large prospective cohort study in the South West of England [8] showed that four year incidence of obesity was higher between the ages of seven and 11 years than between 11 and 15 years, suggesting that mid childhood may be an appropriate age in which to deliver prevention programmes. 

When designing an intervention to affect children’s behaviours it is important to try and impact both the home and the school environment as these are important influences on a child’s eating and physical activity behaviours [9]. The organisational, social and communication structures in place within schools make it the ideal location to deliver a programme of activities that reach and support children and families across the social spectrum [10]. An intervention has the potential to channel lifestyle messages through the children into the home if designed and delivered in an appropriate and engaging way that is mindful of the home environment. In addition, teachers and head teachers are generally supportive of such approaches as they not only promote the wellbeing of the child but also provide opportunities for schools to engage with parents [11]. Consequently, health researchers and guidelines all highlight the role of schools in obesity prevention [12,13]. However, there are challenges associated with school-based research; teachers and administrators are very busy, thus, although they might be supportive of the study, engaging them sufficiently to “buy in” and “run with” a programme can be difficult. We believe that achieving this school engagement is crucial to creating sufficient interest in a school to draw parents in, something that has proven to be a major challenge in previous school-based research [14] and might go some way to explaining the paucity of evidence for effective obesity prevention interventions.

The most promising school based interventions follow the WHO’s Health Promoting School (HPS) framework which advocates a holistic, settings-based approach, consisting of a cycle of steps to guide and implement change in a flexible manner with a focus on action in three areas; the curriculum; the school ethos/environment and links with families/communities [15]. Parents are key to developing a home environment that fosters healthy eating and physical activity. Parents’ knowledge of nutrition; their influence over food selection, meal structure and home eating patterns; their modelling of eating and sedentary habits, including screen time, are all influential in their children’s development of lifelong habits that contribute to normal weight, overweight or obesity [16,17]. Thus involvement of parents must be central to any intervention that aims to affect child behaviours. 

This holistic approach promotes the building of positive relationships with teachers, pupils and families in order to promote a consistent message and develop a sense of “school connection” (a pupil’s sense of feeling part of his/her school, feeling valued and being treated fairly) [18,19] which has been linked to improvement in child health and wellbeing [20]. The importance of the quality of the social relationships and the school as a social institution in promoting child health and wellbeing has long been advocated by Rutter [21] and subsequent research exploring the impact of more specific school and classroom characteristics which influence pupil engagement in learning and social development has shown that student progress is positively associated with good relationships between teachers and pupils, opportunities for pupil participation and responsibility and support structures for teachers [22,23,24,25]. 

Despite this strong rationale for building trusting and supportive relationships at the level of the school, child and family in promoting child health and wellbeing, there has been a paucity of obesity prevention interventions that specifically aim to positively affect relations within the school and within the family in addition to affecting individual level processes, such as improve knowledge and skills and modify norms [26]. A recent review by Khambalia and colleagues [27] examined the findings from existing systematic reviews and meta-analyses of school-based obesity prevention programmes. Unfortunately, most of the studies in the reviews examined did not report on the specifics of intervention components or the context in which they had been delivered and the heterogeneity of the studies made it difficult to draw conclusions. The most recent Cochrane review [14] found that of the 39 trials targeting primary school-aged children, one third did not report any process measures. As a result Khambalia and colleagues were unable to make any clear guidelines in relation to specific programme characteristics predictive of success and could only conclude that interventions associated with a significant reduction of weight in children were of long term duration, focused on both diet and physical activity and included a family component. 

In this paper we will present an overview of the conceptualisation and development of a novel obesity prevention intervention, the Healthy Lifestyles Programme and implications for future behaviour change interventions. Underpinning the development was the commitment to build relationships at the level of the school, child and family and create opportunities to bring parents into the school and for children to take the messages home to create supportive school and family environments. We will show how this approach has guided the intervention mapping process, involving two pilot studies and an exploratory trial. We will present the phases and components of the resultant intervention and how each phase has been designed to maximize the engagement of families and be feasible and acceptable to schools and children. 

## 2. Conceptualisation of HeLP

A crucial focus for us in developing an obesity prevention programme was to promote engagement of schools, children and their families throughout the intervention, as we believed this to be essential for behaviour change to occur. In line with the HPS framework, we took a whole school approach and aimed to develop activities that impacted the school environment as well as specific behaviours of children and their families. First and foremost we sought to build supportive and trusting relationships by employing deliverers with specific skills and competencies, using the initial phase of the intervention to create a receptive context and by using engaging delivery methods to try and increase the uptake of the programme. Previous research into preventing childhood obesity have found it difficult to engage parents in order to affect change with the family [28,29], thus we believed that the delivery methods within the school environment needed to be sufficiently dynamic, creative and empowering to motivate the children to talk about the activities at home with their parents and encourage them to come into the school to attend key events. Most school-based obesity prevention interventions to date have used traditional delivery methods, such as education lessons to teach children about the importance of healthy nutrition and physical activity as opposed to methods where the child actively engages with the messages [27]. One exception is an intervention that used theatre as a novel delivery approach [30]. This intervention, aimed at low income children and their parents, showed that an after school theatre programme motivated and engaged both parents and children and increased awareness of the need for making changes, however, on its own, was not sufficient to change behaviours. The authors concluded that further development should be made to incorporate this novel delivery method into more comprehensive programmes with both educational and environmental components. In developing HeLP we were mindful that the children themselves, if suffciently motivated, were a key resource in taking messages home to their families, encouraging their parents to attend activities and in affecting change at home, thus interactive drama was a delivery method we explored in the development of HeLP as it showed promise in promoting positive attitudes towards a number of health behaviours [31] and was a means of delivering a range of behaviour change techniques. 

It was agreed that each phase of the intervention should be defined in terms of its “function” (the overall aim of each phase) as well as its “form” (individual components within each phase) and that components within each phase could be adapted slightly to better fit the context in which it was being delivered. Early piloting work [32,33] enabled us to understand the school culture, resources, constraints and capacity and thus what parts of the intervention could be open to adaptation whilst still remaining true to the programme. In the development phases we were also mindful to minimise the potential to widen existing health and social inequalities and to ensure that the intervention was feasible and accessible to schools, children and their families from all socioeconomic backgrounds/status. 

From the outset we wanted to develop a programme which affected both the upstream and downstream influences on health behaviours as well as build relations to affect family behaviours [26]. The underpinning assumptions were to create supportive environments for healthy lifestyles choices and positive relationships at the level of the school, child and family. Thus HeLP was conceptualised as a complex intervention within a complex system and developed as a dynamic, evolving set of processes with re-enforcing feedback loops between the school, child and family [34]. It was believed that individual components within the programme continually interacted with each other over time to achieve the desired outcome, and that the deliverer was part of the intervention. Hence, we have not sought to determine whether some components were more effective than others, so-called “active ingredients”, rather we have characterised the components in terms of their functions and the relational qualities necessary for effective delivery. 

## 3. The Development of the Healthy Lifestyles Programme (HeLP)

We used Intervention Mapping (IM) [35] to develop the intervention as it combines multiple theoretical and experiential perspectives with new research to assess and/or develop a potential set of possible solutions for a particular problem rather than defining a practice or research agenda around a single specific theory [36]. A substantial amount of time in the first step of IM (needs assessment) was spent engaging teachers and the local education and health authority to understand the primary school system and how best to work with schools to deliver a programme of activities related to healthy lifestyles which children and their families would want to participate in. In order to affect individual level processes and define possible behaviour change techniques (BCTs) to employ within the intervention, we used the Information, Motivation and Behavioural Skills Model (IMB) as a guide [37]. This model was chosen as it linked closely to the selected determinants of our three key behavioural objectives; reducing sweetened fizzy drink consumption; increasing the proportion of healthy to unhealthy snacks consumed and reducing sedentary behaviours. In order to promote children’s access and availability of opportunity (a key determinant not considered in the IMB model), we sought to engage parents and offer them strategies through which they could directly (through parenting) or indirectly (through the creation of supportive environments) foster the development of healthy eating and activity behaviours among their children/family. In order to guide the sequential order in which the behaviour change techniques were to be delivered, we used the Health Action Process Model (HAPA) as a guide [38]. This phased model implies a clear order of distinct actions starting with establishing motivation, moving on to taking action followed by maintaining motivation. 

### The HeLP Intervention

The Healthy Lifestyles Programme (HeLP) consists of four ordered phases (based on the HAPA model described above) each with multiple components and a range of delivery methods (see Table 1). The intervention targets the year 5 children (although some components are also delivered to the whole school) and runs over three school terms (spring and summer term of year 5 and autumn term of year 6). The aim is to deliver a general healthy lifestyle message encouraging a healthy energy balance with a focus on changing three specific behaviours relating to energy intake and expenditure; decreasing the consumption of sweetened fizzy drinks; increasing the ratio of healthy to unhealthy snacks consumed, and reducing screen-based activities*.* We adopted the “80/20” mnemonic, which suggests we should be active and eat healthily 80% of the time. This “tag” came out of the early pilots as parents and children found that it acted as a trigger for remembering the three key behaviours. 

**Table 1:** Intervention phases, function, BCTs and the component and agent of delivery.

Intervention Phase	Function	Behaviour Change Techniques (BCTs)	Component (Frequency and Duration)	Agent of Delivery
**Phase 1** **Creating a supportive context** Spring term (Year 5) January–March	Establish relationships with schools, children and families Raise awareness and Increase knowledge Promote positive attitudes and norms towards healthy eating and physical activity Increase self-efficacy for behaviour change	Provide information on behaviour-health link Provide information on health behaviour link Modelling/demonstrating behaviour Prompt identification as a role model Provide information on behaviour-health link Skill building	Whole school assembly (1 × 20 min) Newsletter article Literacy lesson (to create HeLP rap-poem) (1 × 1 h) Activity workshops (2 × 1.5 h) Parent assembly (1 × 1 h) involving child performances	HeLP Coordinator (HC) HC Class teacher Sports teams/dancers Class teachers/HC/Drama group
**Phase 2** **Intensive Healthy Lifestyles Week—**one week Summer term (Year 5) April–June	Strengthen relationships with schools, children and families Increase knowledge Increase self-awareness Increase self-efficacy Develop communication and problem solving skills Increase social support (school, peer and family)	Provide information on health behaviour link Problem solving/barrier identification Modelling/demonstrating behaviour Prompt identification as a role model Communication skills training Teach to use prompts and cues	Education lessons (5 × 1 h) (morning) Drama (5 × 2 h) (afternoon) (forum theatre; role play; food tasting, discussions, games* etc.*).	Class teacher Drama group
**Phase 3** **Personal Goal Setting with Parental Support-** goals set during week following drama Summer term (Year 5) June–July	Increase awareness of own behaviour Increase self-efficacy for change Develop planning skills Increase parental support	Self-monitoring Goal setting (behaviour) Problem solving/barrier identification Plan social support Provide information on where and when to perform a behaviour Agree behavioural contract Prompt identification as a role model	Self-reflection questionnaire (1 × 40 min) Goal setting sheet to go home to parents to complete with child (1x10 mins) 1:1 goal setting interview (1 × 10 min) (goals sent home to parents) Forum theatre assembly (1 × 1 h)	HC HC and Parents HC HC/Drama group
**Phase 4** **Reinforcement Activities** Autumn term (Year 6) September–December	Increase self-awareness and prioritise healthy goals. Consolidate social support. Develop self-monitoring and coping skills Increase parental support	Provide information on health behaviour link Modelling/demonstrating behaviour Prompt identification as a role model Provide social approval Prompt self-monitoring Prompt intention formation Follow up prompts Prompt review of behavioural goals Prompt barrier identification and resolution Coping plans	Education lesson (1 × 1 h) Drama workshop (1 × 1 h). Followed by a class delivered assembly about the project to rest of school (1 × 20 min). 1-to-1 goal supporting interview to discuss facilitators/barriers and to plan new coping strategies (1 × 10 min) (renewed goals sent home to parents)	Class teacher Drama group HC HC

Phase 1, *Creating a Supportive Context* aims to establish relationships, and raise awareness of HeLP, setting the foundation for the successful delivery of subsequent components. Professional sports people and dancers are used to talk to the children about the importance of healthy lifestyles and run practical workshops. This creates a buzz in the school and sets a positive atmosphere for future activities. Children then showcase the skills they learn during these workshops in a parent assembly at the end of phase 1, where they are given further information about the programme by the HeLP Coordinator (HC). Phase 2 is the intensive *Healthy Lifestyles Week* involving education lessons (delivered by the class teacher) and interactive drama activities (delivered by a local drama group). The drama framework includes four characters (Disorganised Duncan, Fooball Freddie, Snacky Sam and Active Amy), each represented by one of the actors, whose attributes related to the three key behaviours. Children choose which of the characters they most resemble then work with that actor to help the character learn to change their behaviour. It was believed that this strategy would promote greater ownership of the messages and lead to increased intrinsic motivation for the goal setting [39] which occurs in phase 3. A technique we use during these sessions is Forum Theatre where actors act out a family scene and children must focus on the behaviours of one character. If they notice the character not adhering to the healthy lifestyle messages the children can shout “stop” and suggest the change the character could make to improve the outcomes. The child then enters the scene taking on the role of the character and the scene is rerun with the suggested change. This method brings the children into the performance enabling them to have an input into the dramatic action they are watching. Such a technique enables the children to explore possible solutions and propose real changes in preparation for real life situations, bestowing a sense of empowerment [40]. Phase 3 is *Personal Goal Setting with Parental Support* and encourages the children to reflect on their own behaviours and set goals (based on the HeLP messages) with their parents. Phase 4 is *Reinforcement Activities* and involves a range of components to refocus the children and their parents on the HeLP messages and behaviour change. Table 1 shows each phase of HeLP, its function, the BCTs used and the component (form) and agent of delivery. 

#### Parental and School Engagement

Each phase of HeLP has been designed to involve parents as much as possible; in phase 1 there is a newsletter and parent assembly. In phase 2, an information leaflet goes home to parents each day based on the theme covered in the drama session and parents are invited in to the school to watch work in progress during the last two drama sessions of the week. In phase 3, parents set goals at home with their child, which are written up and sent home along with the HeLP “80/20” fridge magnet. Following this, there is another parent assembly. In phase 4, following the one on one goal-supporting interview, the children’s goals are, once again, sent home in the post. We were mindful that we also wanted to affect the whole school environment, thus certain components within each phase are directed to the whole school e.g., there is a whole school assembly in phase 1 and a class delivered assembly to the whole school in phase 4. The intervention has been designed so as to enthuse the staff such that they will want to adopt the messages and continue with related activities during or after the intervention. The class teacher is specifically tasked with delivering the Personal Social and Health Education (PSHE) lessons prior to the interactive drama during the Healthy Lifestyles Week (Phase 2) and a literacy rap lesson during Phase 1. We wanted the teachers to deliver some aspects of the programme so that they were more likely to engage with HeLP and become familiar with its content; such that they would be able to use it in other aspects of their teaching if they so wished. We have ensured that all lesson plans link to National Curriculum objectives for PSHE and these have been manualised in a booklet with all associated resources on a CD Rom. All worksheets for these lessons are photocopied and prepared in advance by the HC. Our hypothesis is that school engagement will strengthen child, and thus parental, engagement with the programme.

#### Delivery of HeLP

As Table 1 shows, HeLP is delivered by a combination of personnel. Piloting of the intervention in the early stages identified the actors and the HeLP Coordinator (HC) as key to programme delivery. The role of the HC is to build trusting and supporting relationships, which requires specific competencies, understanding and interpersonal skills such as the ability to listen reflectively and empathise with children, parents and teachers. It was believed that one key contact person for each school was crucial in building and strengthening relationships with teachers, children and parents over the course of the one year intervention and would lead to the engagement with the programme believed to be crucial to behaviour change. The HeLP Coordinator, therefore, is as much a part of the intervention as the components within the programme. HeLP was designed to be delivered in a collaborative manner, promoting identification with and ownership of the healthy lifestyle messages so that the children are motivated for autonomous reasons [39]. We believed this would create the necessary conditions for change at both a child and family level. The actors who deliver the interactive drama workshops are highly skilled in the drama techniques used, having completed a week of intensive training prior to delivery of the Healthy Lifestyle Week. All the activities and scripts for this week have been manualised. Similarly, the HC practise the delivery of the whole school assembly and parents’ evenings in front of our stakeholder group which was created during the piloting of HeLP (see Section 4).

Whilst all components have been manualised so that delivery is standardised, HeLP has been designed to allow for some flexibility so that each activity can fit the context of the school, for example, schools are able to select the timings of parent assemblies, which may occur in the morning or at the end of the school day. The HC works closely with teachers to understand how best to engage and involve the parents, which can vary depending upon the type of school.

The Intervention Mapping process, discussed earlier, requires intervention developers to break down their key behavioural objectives into smaller steps (performance objectives) so that specific and appropriate BCTs and strategies to implement these can be selected. Table 2 and Table 3 show the performance objectives we produced based on our three overall behavioural objectives (reducing sweetened fizzy drink consumption; increasing the proportion of healthy to unhealthy snacks consumed; and reducing sedentary behaviours) and their associated BCTs and implementation strategies. These have been mapped onto the three processes of behaviour change outlined in the HAPA model (establish motivation, take action, maintain motivation), which guided the sequencing of the HeLP Programme (Table 1) and provides a more detailed view of specific intervention activities for the reader.

**Table 2:** “Establish Motivation”: associated behaviour change techniques and strategies for performance objectives.

Performance Objectives	Behaviour Change Techniques	Implementation Strategies
**A** Communicate healthy lifestyle messages to parents and seek their help and support	Exchange information Prompt barrier identification Model/demonstrate behaviour Communication skills training Prompt identification as a role model	Children learn about the healthy lifestyle messages and support strategies through a variety of individual and group tasks delivered by the teacher in PSHE lessons and by actors in drama workshops. “80/20” used as a general message throughout suggesting we should eat healthily and be active at least 80% of the time. Parent information sheets given to children following each drama workshop. Characters and children role-play scenes to communicate messages to parents and seek their support. Discussion and role-play of ways to encourage whole family to make changes. Characters present scenes, where after having made changes to their behaviours, become role models to others (siblings, parents, friends) followed by group discussion
**B** Select and try healthy alternatives to unhealthy snacks and drinks at home and at school	Exchange information Provide encouragement Modelling	Children view and discuss with their chosen character ingredients of both healthy and unhealthy food and drink. Compare fat, sugar and salt content to recommended guidelines. Children observe characters taste healthy snacks and drinks while role playing in different settings Characters provide encouragement Children taste healthy snacks and drinks with their chosen character
**C** Select feasible active alternatives to sedentary activities	Modelling	Children and actors role play home and school scenes focusing on replacing sedentary leisure pursuits with active alternatives. Children play interactive games to choose and mime active leisure pursuits. Children observe characters mime 24 h clock and discuss their activity in relation to 80/20 message
**D** Understand and resist temptation	Prompt barrier identification Problem solving Decision balance Prompt barrier identification Model/demonstrate behaviour Communication skills training	Children make personalised “Temptation T shirts” Children work with their chosen character to prepare ways to tempt the other 3 characters and help their own character to resist temptation. Children participate in the “Temptation Ladder” activity that enables them to practise skills to resist temptations and help others. Children observe characters role play marketing scenes Children participate in the role-play.

**Table 3:** “Take Action” and “Stay Motivated” associated behaviour change techniques and strategies for performance objectives.

Performance Objectives	Behaviour Change Techniques	Implementation Strategies
**E** *(Take Action)* Reflect on own snacking and leisure choices	Raising awareness Prompt intention formation	Children reflect on snacking and leisure choices in individual and group classroom tasks as well as homework tasks Children complete step 1 (self-reflection) of the “goal setting sheet”
**F** *(Take Action)* Set goals and make changes	Implementation intentions Prompt social support from family Prompt specific goal setting Teach to use prompts or cues Coping plan Model/demonstrate behaviour Prompt identification as a role model	For each goal (set with parents) children write what strategies they can use to help with goal achievement In presence of parents, children write down the support they need to achieve their goals (step 3 of “goal setting sheet”) Children have one to one discussion about agreed goals, possible barriers and coping strategies with researchers (goals and strategies sent directly home to parents) Children given pedometer as a motivational tool Children participate in Forum Theatre Parents observe/participate in Forum Theatre
**G** *(Stay Motivated)* Monitor goals	Prompt self-monitoring of goals	Children produce a personalised self-monitoring chart in class (taken home with letter to parents). A copy of this chart is kept in school for children to look at and complete e very three weeks
**H ***(Stay Motivated)* Assess barriers to goal achievement	Prompt review of behavioural goals Prompt barrier identification Coping plan	Group and individual class activities to assess facilitators and barriers to goal achievement Children observe and participate in scenes with characters to role play barriers experienced One to one goal supporting interview with researcher to discuss facilitators/barriers to goal achievement and plan new coping strategies to aid goal achievement
**I** *(Stay Motivated)* Adapt goals	Prompt intention formation	Children agree adapted goals with researcher and parents (new goals and strategies sent home to parents)

## 4. Piloting

Early development and evaluation of HeLP involved two pilot studies involving 200 children. The aim of the first pilot was to trial different methods to deliver the selected behaviour change techniques to promote the healthy lifestyle messages across three age groups in one primary school. The aim of the second pilot was to refine the intervention based on pupil, teacher and parent responses from pilot one and carry out a before and after study looking at behavioural outcomes in another primary school [32].

Key findings to emerge from the data in the first pilot were that the year 5s (9–10 year olds) were most receptive to the messages and engaged their parents to the greatest extent and that it was more feasible for the school to run the HeLP activities in year 5 rather than year 6. Similarly, although the younger age group (8–9 year olds) enjoyed the programme, they were less able to take the messages home to their parents. As a result, year 5 was selected as the target group. The children were unanimous in their enjoyment of the drama activities, with many parents reporting that their child had talked about the activities at home, encouraged other family members to make changes and wanted their parents to come into the school to view programme activities. Another outcome of pilots 1 and 2 was the creation of a project stakeholder group consisting parents, teachers and healthy lifestyle advisors working in Public Health who were willing to advise us on further development of the intervention and the trial design. The results from these pilots are published in detail elsewhere [32,35].

### The Exploratory Trial 

Pilot 3 was an exploratory randomised controlled trial which sought to assess for schools, children and their families; recruitment and retention in control and intervention schools; feasibility and acceptability of HeLP; feasibility and acceptability of future trial outcomes and facilitators and barriers to the uptake of HeLP. Full results have been published elsewhere [33,41]. In summary, the trial was conducted with four schools, including seven Year 5 classes and 204 children. Baseline measures (height, weight, waist circumference and body fat, objectively measured physical activity, food consumption using the Food Intake Questionnaire [42] and TV/screen time, assessed using TV viewing habits questionnaire [43]) were taken on 202 children who consented to participate. Schools were then randomised to receive the HeLP Programme or to be control schools. The measures were repeated at 18 months, on all children, when they were in Year 6 and height and weight, waist circumference and body fat measures were taken again at 24 months when the children had moved to secondary school. The results showed that schools, children and their families found the trial design and the intervention feasible and acceptable with only three children not consenting to participate in the research. Over the course of the study a further eight children were lost to follow up (three withdrew and five moved out of the area). At 18 months follow-up, intervention children had fewer negative food markers, consumed less energy dense snacks and more healthy snacks, had more positive food markers, had lower mean TV/screen time and spent more time doing moderate to vigorous physical activity each day than children in the control schools. The percentage of children classified as overweight or obese at baseline was similar for both intervention and control at baseline (24% and 26%, respectively) but this had increased to 32% at 18 and 24 month follow ups in the control children whilst remaining at baseline levels in the intervention children. Overall, intervention children had lower anthropometric measures at 18 and 24 months than control children, with larger differences at 24 months than at 18 months for all measures except percentage body fat summed difference score [41]. 

In 2012 funding was secured from NIHR-PHR to conduct a 32 school randomised controlled trial (involving 1324 children) of HeLP to determine its effectiveness and cost effectiveness in preventing childhood obesity [44]. To date, we have 98% follow up at 18 months and 93% follow up at 24 months in our first cohort of 16 schools. Full results will be available in October 2016.

## 5. Implications for Future Behaviour Change Programmes

We sought to develop an obesity prevention programme, which could engage schools, children and their families sufficiently to affect obesity related behaviours at both a child and family level. Based on the development of HeLP and the three pilot studies [32,33,35,41] we believe that the key aspects of HeLP that lead to this engagement are;

- **Having one key contact person (the HeLP Coordinator) who has the necessary skills and competencies to build relationships with the schools, children and their families. **
  During early pilot work it became apparent that, in order for HeLP to be delivered as designed it was crucial to employ personnel with the qualities necessary to engage school staff, children and their families. This led the development team to consider, in depth, the necessary qualities required of the role and how best to assess these during recruitment for the definitive trial. In addition, experiential learning has enabled us to produce a detailed training manual for the HeLP Coordinator role, ensuring that delivery is of the quality necessary to engage sufficiently to affect behaviour change.
- **Using highly interactive drama activities within a framework based on four identifiable characters played by well-trained young actors.**
  Techniques used in the drama sessions allowed children to co-create scenes with the actors and, as such, based learning on the dialogic relationship between fiction and reality, which allows rehearsal for real life [45]. The Confucian aphorism; “tell me and I will forget”, “show me and I may remember” and “involve me and I will understand” is particularly relevant when choosing delivery methods to engage children with healthy lifestyle messages so that they are motivated to take them home to their parents and discuss them with their peers.
  Despite its potential to empower and engage children, only a few health promotion programmes have primarily or solely involved drama methods in school-based health promotion programmes [46,47,48]. One reason for this could be that most teachers have only limited experience and therefore lack the ability and confidence to use drama methods [49]. Data from the pilot phases show that quality delivery by those who have the skills is essential and that identification with the four characters played by young actors is the means by which trusting relationships and a bond with the actors can be developed.
- **Ensuring that key components of the programme were delivered by outside personnel.**
  Stakeholder consultation in the early development phases revealed that primary school teachers do not have the time to attend training sessions in order to deliver such programmes. As HeLP is delivered, in the main, by people external to the school, it does not require teachers to attend training sessions and this was a strong reason for its feasibility and acceptability amongst school staff. This was also something, which was “valued” by the children as it meant the programme “did not feel like school”.
- **Creating an intervention that can be adapted to the context of each school, whilst still remaining true to HeLP.**
  To enable adaptability, HeLP has been defined both in terms of “form” (each component and their specific order) as well as “function” the intended purpose of each phase. This allows the HC and the teachers to work together to adapt some activities so that they are able to run within the context of that particular school, whilst still achieving the aim of the activity. For example, it is not feasible to run the class delivered assembly in phase 4 in the traditional format with the large schools (circa 3 years groups per year) due to limited space, so in consultation with teachers and the HC the format was adapted to small groups of year 6 children going in to different year group classes to deliver the 15 min “performance” rehearsed in the drama workshop.
- **Devoting a whole phase of the intervention to build trusting and supportive relationships.**
  It was believed that a necessary condition for behaviour change to occur was the building of trusting and supportive relationships and that the successful delivery of phase 2–4 was dependent upon the HeLP Coordinator getting to know the teachers, children and their parents as well as understanding the running of the school. Activities in this phase were chosen to allow time for relationships to develop and to create a sense of excitement within the school about the activities to follow.
- **Developing an intervention that meets National Curriculum objectives for Key Stage 2. **
  Data from the pilot work showed that teachers thought that the programme was compatible with the National Curriculum and that there were many opportunities to make links to other subject areas. This increased feasibility to deliver the components and thus promoted engagement at the level of the school.
- **Active and on-going involvement of the project stakeholder group.**
  The formation of a stakeholder group, which had continued to grow as we have progressed through the piloting phases, has been an invaluable source of advice and information. The group have guided the research team in the wording of questionnaires for parents and children, helped to recruit the HeLP Coordinators, allowed their contact details to be given to parents who might like to talk to someone other than the HC or the Trial Manager about the programme and provided critical feedback to the HCs during practise delivery of the parent assemblies.

## 6. Conclusions

In summary, the Healthy Lifestyles Programme is highly innovative and novel, not only in the delivery methods used, but also in how it has been conceptualised. We believe that in order for behaviour change to occur in school-based programmes, it is essential that they are viewed as a dynamic, evolving set of processes with feedback loops between the school, child and family within which the building of trusting and supporting relationships is a necessary condition for behaviour change to occur. Within the process evaluation of the definitive trial of HeLP, we are seeking to understand the level of engagement of schools, children and their families, which will not only provide information on how the intervention may be working but will also inform future behaviour change interventions. 
